# Novel Electrochemical Sensor Fabricated for Individual and Simultaneous Ultrasensitive Determination of Olaquindox and Carbadox Based on MWCNT-OH/CMK-8 Hybrid Nanocomposite Film

**DOI:** 10.3390/molecules24173041

**Published:** 2019-08-22

**Authors:** Yanqing Liu, Gengxin Hu, Hongwu Wang, Su Yao, Yinjian Ye

**Affiliations:** 1 School of Food & Pharmaceutical Engineering, Zhaoqing University, Zhaoqing 526061, China; 2 Guangdong Engineering Technology Research Center for Food & Agricultural Product Safety Analysis and Testing, Zhaoqing 526061, China; 3 School of Chemistry and Environment, South China Normal University, Guangzhou 510006, China

**Keywords:** CMK−8, MWCNTs−OH, electrochemical sensor, differential pulse voltammetry, simultaneous determination, carbadox, olaquindox

## Abstract

A hybrid nanocomposite consisting of hydroxylated multi-walled carbon nanotubes (MWCNTs−OH) and cube mesoporous carbon (CMK−8) was applied in this study to construct an MWCNT−OH/CMK−8/gold electrode (GE) electrochemical sensor and simultaneously perform the electro-reduction of olaquindox (OLA) and carbadox (CBX). The respective peak currents of CBX and OLA on the modified electrode increased by 720- and 595-fold relative to the peak current of GE. The performances of the modified electrode were investigated with electrochemical impedance spectroscopy, cyclic voltammetry, and differential pulse voltammetry. Then, the modified electrodes were used for the individual and simultaneous determination of OLA and CBX. The fabricated sensor demonstrated a linear response at 0.2–500 nmol/L in optimum experimental conditions, and the detection limits were 104.1 and 62.9 pmol/L for the simultaneous determination of OLA and CBX, respectively. As for individual determination, wide linear relationships were obtained for the detected OLA with levels of 0.05–500 nmol/L with LOD of 20.7 pmol/L and the detected CBX with levels of 0.10–500 nmol/L with LOD of 50.2 pmol/L. The fabricated sensor was successfully used in the independent and simultaneous determination of OLA and CBX in spiked pork samples.

## 1. Introduction

Veterinary medicine is indispensable in the animal feeding development and breeding industries. Quinoxalines, a heterocyclic compound with benzene and pyrazine rings, can destroy and inhibit the synthesis of bacterial DNA, and it can improve feeding efficiency, animal growth, and antibacterial efficacy [1,2,3,4]. Carbadox (CBX) and olaquindox (OLA) are classical quinoxalines and used widely in swine feeds to promote growth, increase the rate of weight gain, and prevent dysentery and bacterial enteritis in young swine [5,6,7,8]. Thus, CAB and OLA have been considerably used in the last century. However, the excessive use of quinoxalines has led to drug residue accumulation and high drug resistance. Given the health concerns on possible photoallergenicity, mutagenicity, and carcinogenicity, many countries and regions have focused their efforts on regulating CAB and OLA usage. Since 1998, the European Commission has withdrawn both drugs from the market because of their possible carcinogenic and mutagenic effects [8,9,10,11]. OLA is also prohibited in USA, Brazil, and Mexico.

The use of OLA in China is only allowed as a medicated premix for pigs with weights less than 35 kg, and it is currently banned in poultry and aquatic breeding. The use of CBX is permitted in many countries, including USA, but it is banned in China [5,12,13]. Despite strict control, CBX and OLA have been used excessively and frequently misused. Therefore, a sensitive and reliable method to monitor CBX and OLA residues in animal feeds is urgently needed.

Many methods, such as high-performance liquid chromatography (HPLC) [14,15,16], HPLC with mass spectrometry [17,18,19,20], gas chromatography mass spectrometry [12], and immunosorbent assay, have been developed to detect quinoxalines and their metabolites [9,21,22]. However, these methods require expensive equipment, complicated and time-consuming sample preparation processes, and highly skilled personnel and technicians, and thus are limitedly applied. Moreover, the methods are unsuitable for onsite detection.

Electrochemical sensors (ECSs) are highly sensitive, specific, rapid, and inexpensive, and they are capable of fast response and miniaturization; thus, ECs have been widely used in the monitoring of residues [23,24,25,26], including OLA [27,28,29,30]. However, no studies have been reported on the use of electrochemical methods for CBX or the simultaneous determination of CBX and OLA.

Ordered mesoporous carbon (OMC) materials have recently attracted considerable attention owing to their well-ordered pore structures, highly specific surface areas, good pore volume, narrow pore size distribution, good stability, and high electric conductivity [31], which are crucial factors in many applications. Since the first synthesis of OMC in 1999 [32], it has been extensively used an as adsorbent, catalyst, and supercapacitor, and applied in energy storage and conversion [33,34,35,36]. OMC is usually synthesized by template methods. Different mesoporous carbon materials can be obtained by changing the pore structures of mesoporous silicon templates. However, the research on OMCs, especially the application of cube mesoporous carbon (CMK−8) in ECSs, is currently insufficient.

In this work, hydroxylated multi-walled carbon nanotubes (MWCNTs−OH) and a CMK−8 nanocomposite-modified electrode were used to fabricate a novel ECS. The fabricated ECS exhibited excellent catalytic activity for the electro-reduction of OLA and CBX. The respective peak currents of OLA and CBX on the modified electrode increased by 720- and 595-fold relative to the current peak of a gold electrode (GE). ECS was successfully used for the simultaneous detection of trace CBX and OLA in a complex matrix.

## 2. Results and Discussion

### 2.1. Characteristics of Materials

**Figure 1:**
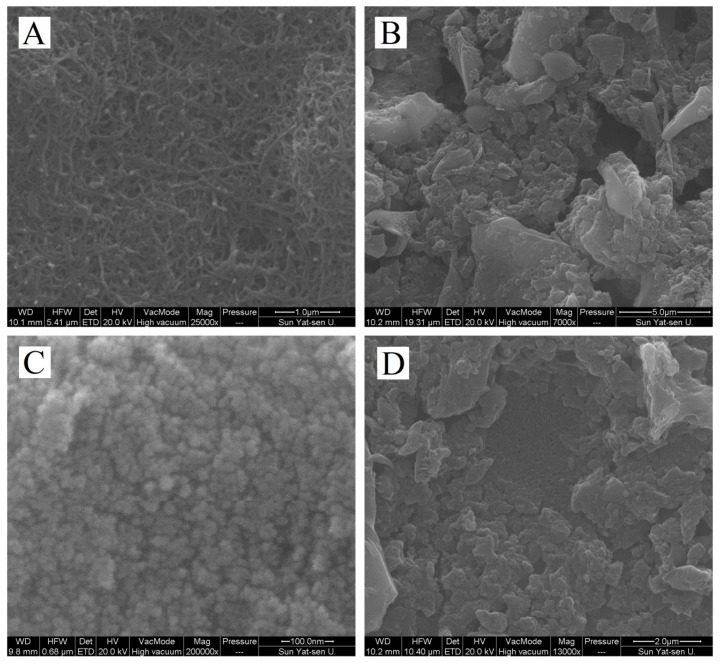
SEM images of MWCNTs−OH (**A**), CMK−8 (**B** and **C** for low and high magnifications, respectively) and MWCNTs−OH/CMK−8 (**D**).

The morphologies of the MWCNTs−OH, CMK−8, and MWCNTs−OH/CMK−8 were recorded by SEM (Figure 1). The MWCNTs−OH exhibited a well-dispersed curly filament morphology with pipe diameter of 50 nm and length of 1−3 μm. The CMK−8 sheets with sizes of 1–5 μm that were stacked onto the modified electrode exhibited several micropores on the surface (10−20 nm). The morphology of the modified MWCNT−OH/CMK−8 electrode is shown in Figure 1D. The CMK−8 layer covered the MWCNTs−OH layer, but some MWCNTs−OH were exposed.

### 2.2. Electrochemical Characteristics

**Figure 2:**
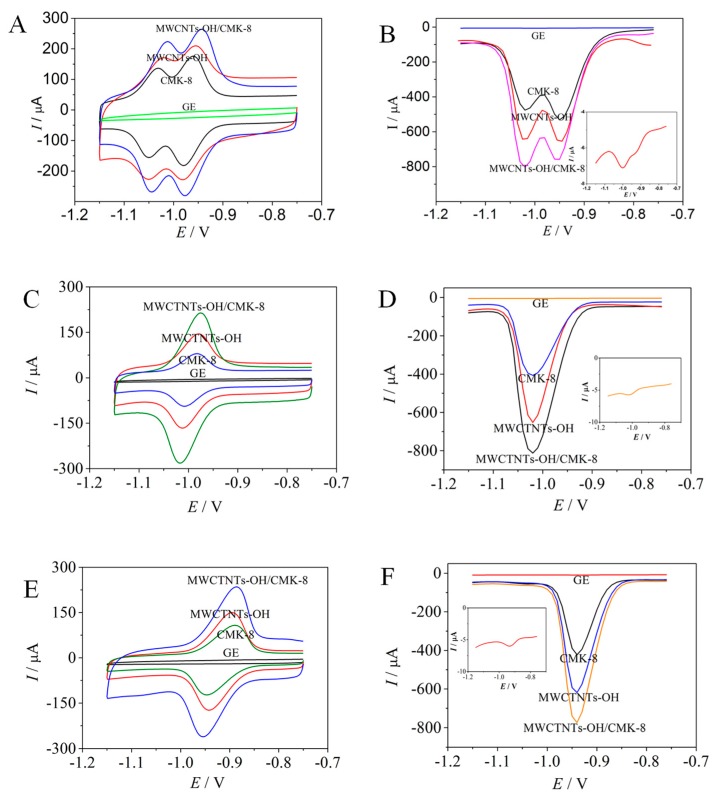
CVs (**A**) and DPVs (**B**) of 1.0 μmol/L OLA and CBX on modified electrode. CVs (**C**) and DPVs (**D**) of 1.0 μmol/L OLA on modified electrode. CVs (**E**) and DPVs (**F**) of 1.0 μmol/L CBX on modified electrode. The inserted graph is the enlarged DPV curve of the GE. The scanned buffer was 0.6 mol/L of Na_3_PO_4_.

CV and DPV were used to characterize the electrochemical behaviors of OLA and CBX (1.0 μmol/L) with different electrodes. The redox peaks of OLA and CBX on the GE were hardly observable (Figure 2). The redox peak currents increased remarkably when the GE was modified with MWCNTs−OH or CMK−8. The increase indicates that MWCNTs−OH or CMK−8 can enlarge an electrochemical surface area and accelerate electron transfer. With the assembly of MWCNTs−OH/CMK−8, the redox peak current continuously increased. The MWCNTs−OH/CMK−8 also further enlarged the effective surface area of the electrode.

The OLA and CBX on the GE (Figure 2B) obtained extremely weak reduction signals at −1.02 and −0.95 V, with peak currents of 0.908 and 1.143 μA, respectively. By contrast, the OLA and CBX on the modified CMK−8 electrode obtained remarkable reduction peaks, with peak currents of 381.8 and 509 μA, respectively. On the modified MWCNT−OH electrode, the OLA and CBX obtained remarkable reduction peaks, with peak currents of 563.2 and 588.9 μA, respectively. On the modified MWCNTs−OH/CMK−8/GE, the OLA and CBX obtained remarkable reduction peaks, with peak currents of 653 and 680 μA, respectively.

Relative to the peak current of GE, the peak current of OLA and CBX on the modified MWCNTs−OH/CMK−8/GE increased by approximately 720- and 595-fold, respectively. Thus, the MWCNTs−OH/CMK−8/GE, which possess strong OLA and CBX electrocatalytic properties, can be used for simultaneous ultrasensitive detection.

### 2.3. Electrochemical Impedance Spectroscopy 

**Figure 3:**
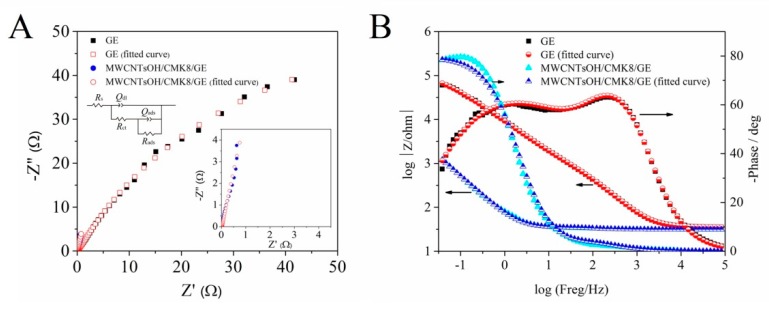
(**A**) Nyquist and (**B**) Bode diagrams of GE and MWCNTs−OH/CMK−8 modified electrode in 1.0 μmol/L OLA. The inset is the Equivalent circuits.

Electrochemical impedance spectroscopy (EIS) was used to evaluate the interfacial properties of the differently modified electrodes. The impedance spectra in Figure 3 correspond to the Nyquist and Bode plots of GE and MWCNTs−OH/CMK−8/GE, with polarization potential of −0.7 V and frequency from 105 to 0.01 Hz in the solution containing 1.0 μM of OLA. The equivalent circuit is in good agreement with the Nyquist curve, as shown by the inset of Figure 3A. In the figure, *Rs* refers to the resistance of the solution, *R*_ct_ represents the resistance of the electron transfer, *Q*_dl_ is a constant phase element corresponding to double-layer capacitance, and *Q*_ads_ and *R*_ads_ are the electronic elements associated with the adsorption of reaction intermediates. The electrode reaction *R*_ct_ in the circuit is an element with a simple physical meaning, i.e., it describes how fast the charge transfer rate varies with the electrode potential in the OLA electro-reduction process when the surface coverage area of the intermediate is unchanged [37]. Then, the equivalent circuit parameters of the impedance spectra were fitted by the Z-view software. The results are listed in Table 1. The *Q*_dl_ of MWCNTs−OH/CMK−8/GE (1.175 × 10^−3^ F) was several orders of magnitude higher than that of GE (6.209 × 10^−6^ F) owing to the enlarged surface area after the combination of the three materials. The MWCNTs−OH/CMK−8/GE obtained a much lower *R*_ct_ (5.884 Ω) than GE (1.176 × 10^3^ Ω), demonstrating the accelerated charge transfer during OLA electro-reduction by using MWCNTs−OH/CMK−8/GE.

### 2.4. Effect of Scan Rate

**Figure 4:**
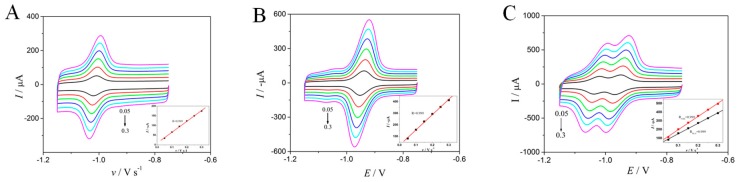
CVs of 1.0 μmol/L OLA (**A**), CBX (**B**), and OLA and CBX (**C**) on MWCNTs−OH/CMK−8 modified electrode with different scan rate from 0.05 to 0.3 V s^−1^, the inset is the dependency of peak current with respect to the scan rate.

The scan rate effects on the electrochemical performances of OLA and CBX were investigated. Figure 4 shows the CV profiles of 1.0 μM of OLA and CBX at the different scan rates between 0.05 and 0.3 V s^−1^. As scan rate increased, the relationship became linear between peak height and scanning rate, indicating the dominance of surface-controlled processes (OLA: *I*p (μA) = 561.3 *v* (V s^−1^) + 8.96, R = 0.999; CBX: *I*p (μA) = 1322.0 *v* (V s^−1^) + 24.8, R = 0.999).

### 2.5. Optimization of Experimental Factors

#### 2.5.1. Influence of Modified Coating Amount

**Figure 5:**
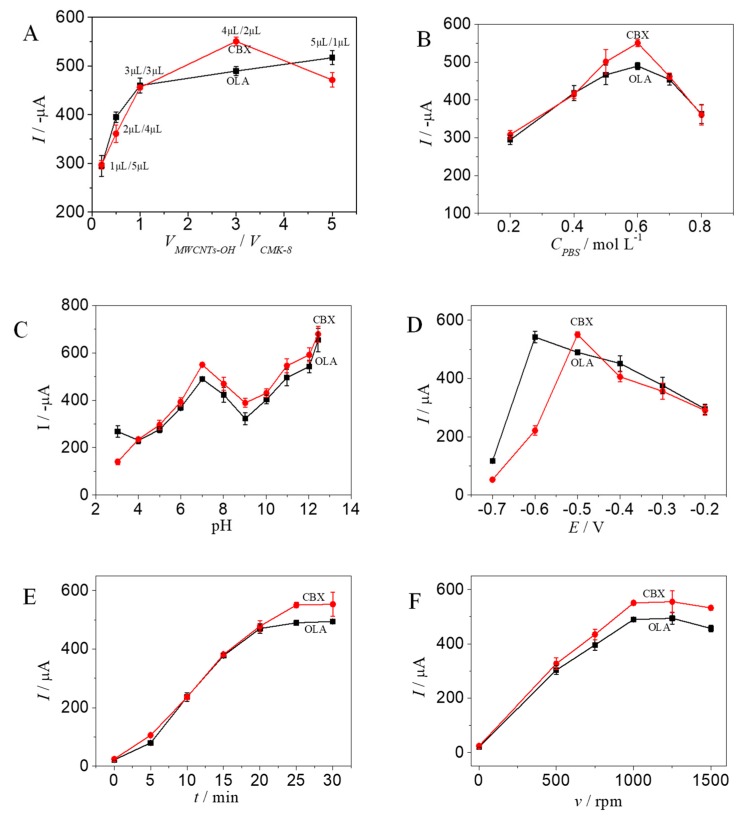
Optimization of the experiment factors (**A**: the volume ratio of MWCNTs−OH and CMK−8, **B**: PBS concentration, **C**: pH, **D**: enrichment potential, **E**: enrichment time, and **F**: stirring speed).

The thickness of the modified electrode film can remarkably affect sensor sensitivity and stability. The peak currents of OLA and CBX increased along with the proportion of MWCNTs−OH (Figure 5A). The peak current of CBX reached the maximum value when the volume ratio between MWCNTs−OH and CMK−8 was set to 4 μL/2 μL. When the proportion was adjusted to 5:1, the peak current of CBX significantly decreased, whereas the peak current of OLA further increased. Therefore, the volume ratio between MWCNTs−OH and CMK−8 was set to 4 μL/2 μL for the simultaneous ultrasensitive determination of OLA and CBX.

#### 2.5.2. Influence of Electrolyte Concentration

The concentrations of the support electrolyte solutions can affect the characteristics of the electrochemical sensors. The current responses of OLA and CBX increased along with PBS concentration (Figure 5B). When the concentration was set to 0.6 mol/L, the response currents of OLA and CBX reached the maximum values. Therefore, the PBS concentration was set to 0.6 mol/L.

#### 2.5.3. Influence of Enrichment Potential

Potential enrichment can significantly accelerate and promote the electro-reduction of OLA and CBX. Thus, the effects of enrichment potential and time were investigated. The modified electrodes were enriched for 25 min at −0.2, −0.3, −0.4, −0.5, −0.6, and −0.7 V and then detected by DPV. The currents of OLA and CBX reached the maximum values at −0.6 and −0.5 V, respectively (Figure 5C). However, the peak current of CBX significantly decreased when the potential was −0.6 V. Therefore, −0.5 V was selected as the enrichment potential for the simultaneous determination of OLA and CBX.

#### 2.5.4. Influence of Enrichment Time

The modified electrodes were enriched at −0.5 V for 0, 5, 10, 15, 20, 25, and 30 min. The peak current increased along with enrichment time and reached the maximum value at 25 min (Figure 5D). The peak current slightly changed when the enrichment time was extended. Thus, the enrichment time was set to 25 min.

#### 2.5.5. Influence of Stirring Speed

Stirring speed affects enrichment efficiency. The peak current gradually increased along with stirring speed and reached the maximum value at 1000 rpm (Figure 5E). Bubbles under high stirring speed were generated and subsequently affected electrochemical reaction stability. Thus, 1000 rpm was selected as the stirring speed.

#### 2.5.6. Influence of pH Measurement

The pH of an electrolyte solution is a primary influencing factor of peak current. The modified electrode was first enriched with OLA and CBX in 0.6 mol/L PBS (pH 7.0), then transferred into a phosphate solution with pH of 3.0–12.5, and finally stirred for 1 s. The peak currents of OLA and CBX reached the maximum values at 12.5 pH (Figure 5F). Thus, the pH value was set to 12.5. Approximately 0.6 mol/L of the Na_3_PO_4_ electrolyte was selected for testing.

### 2.6. Determination of OLA and CBX

**Figure 6:**
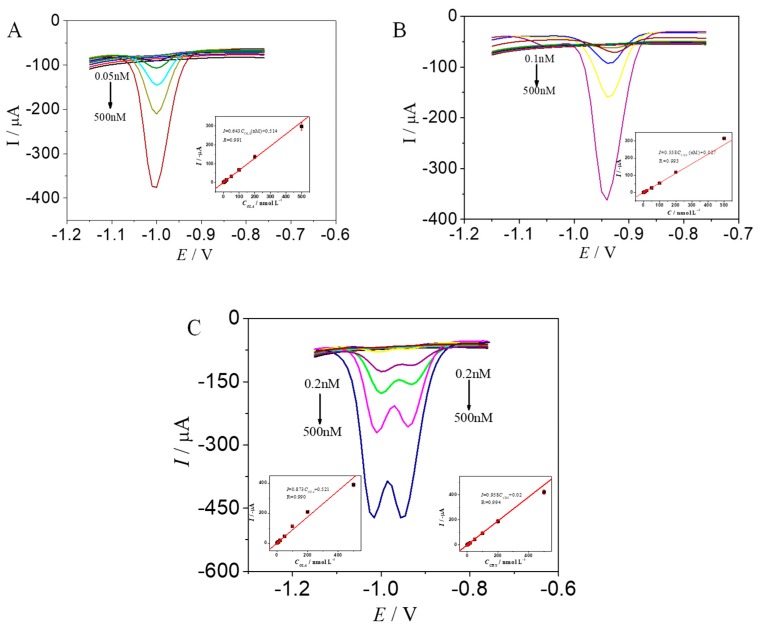
DPVs of different concentrations of OLA (**A**), CBX (**B**), and OLA and CBX (**C**), respectively. The inset shows the resulting calibration curve.

The modified MWCNTs−OH/CMK−8/GE exhibited excellent electrocatalytic performance for the electrochemical reduction of OLA and CBX. Thus, the ultrasensitive electrochemical detection of OLA and CBX can be established. The determination of OLA and CBX was investigated with DPV in optimum conditions (Figure 6).

A good linear relationship was achieved at 0.05–500 nmol/L for OLA (Figure 6A), in which *I* (μA) = 0.514 + 0.643 *C* (nmol/L; R = 0.991). The LOD was 20.7 pmol/L (S/N = 3), and the relative standard deviation (RSD) was 8.02%. 

A good linear relationship was also achieved at 0.1–500 nmol/L for CBX (Figure 6B), in which *I* (μA) = 0.027+ 0.558 *C* (nmol/L; R = 0.993). The LOD was 50.2 pmol/L (S/N = 3), and the RSD was 10.5%.

Figure 6C presents the DPV results with varying OLA and CBX concentrations in the range of 0.2–500 nM. The two detected well-separated cathodal peaks at −1.02 and −0.95 V correspond to the reduction of OLA and CBX, respectively. Approximately 0.07 V of the potential difference between OLA and CBX is regarded sufficient and can be measured simultaneously.

The peak currents represent two good linear relationships for the solution concentration. The regression equations of OLA and CBX were calculated as *I* (μA) = 0.87 C (nmol/L) + 0.52 and *I* (μA) = 0.96 C (nmol/L) + 0.02, and the correlation coefficients were 0.990 and 0.994, respectively. The LOD values were 104.1 and 62.9 pmol/L at S/N = 3.

### 2.7. Repeatability, Reproducibility, and Stability of MWCNTs−OH/CMK−8/GE

The repeatability, reproducibility, and stability of the MWCNT−OH/CMK−8/GE electrode were studied by continuous DPV measurements. The modified electrode was reused seven times sequentially to measure 200 nmol/L of OLA and CBX. No significant changes in DPV response were observed. The RSD of OLA and CBX were 4.25% and 3.76%, respectively. The findings indicate that the proposed sensor has desirable repeatability. Reproducibility was measured on five independent modified electrodes prepared in the same conditions for the detection of 200 nmol/L of OLA and CBX, and the RSDs were 3.75%, and 2.94%, respectively. Thus, the proposed sensor has satisfactory reproducibility. The stability of the proposed sensor was also inspected. No obvious fluctuation was observed for the peak current when the modified electrode was stored at 4 °C for two weeks (7.5% and 5.7% for OLA and CBX, respectively). The results suggest that the proposed MWCNTs−OH/CMK−8/GE have acceptable storage stability.

### 2.8. Interference Study

The interference of the coexisting substances was used to systematically assess the selectivity of the proposed sensor. Urea, glucose, creatinine, uric acid, xanthine, hypoxanthine, and ascorbic acid were 1000-fold but did not exhibit interference on the DPV response to 100 nmol/L of OLA and CBX (peak current change was less than 10%). These results suggest that the sensor has excellent anti-interference ability.

### 2.9. Analytical Application

The applicability of the sensor for OLA and CBX detection was verified by using the MWCNT−OH/CMK−8/GE electrode, particularly by the method of standard addition in pork samples. The experimental results are shown in Table 2. The OLA and CBX recoveries were acceptable and ranged from 96.1% to 107.8%. The RSDs were between 1.11% and 8.18%. The practicability of the fabricated ECS was, therefore, verified.

## 3. Materials and Methods

### 3.1. Chemicals

P123 and TEOS were purchased from Aladdin Inc. (Shanghai, China). *n*-Butanol, HCl, sucrose, and H_2_SO_4_ were obtained from a chemical reagent factory in Guangzhou, China. MWCNTs−OH were provided by Xianfeng NANO (Nanjing, China). OLA, CBX, *N*,*N*-dimethylformamide, sodium dodecahydrate, sodium dihydrophosphate, sodium dihydrophosphate, and sodium hydroxide were provided by Aladdin Biochemical Technology Co. Ltd. (Shanghai, China). All chemicals were of reagent grade quality.

### 3.2. Instrumentation

Electrochemical measurements were performed at an electrochemical workstation operated by Autolab PGSTAT-302N (Metrohm, Utrecht, The Netherlands). The three-electrode system consisted of a platinum wire, Ag/AgCl/KCl electrode, and MWCNTs−OH/CMK−8/GE, which were used as the counter, reference, and working electrodes, respectively. The structures of the materials were characterized by HITACHIS-4800 SEM (Tokyo, Japan) operating at 20.0 kV.

### 3.3. Synthesis of CMK−8

CMK−8 was prepared by using a mesoporous silica KIT−6 as the hare template [38,39]. KIT−6 was synthesized according to a reported method [40], which is a typical synthesis procedure. The molar ratios of P123, TEOS, *n*-butanol, HCl, and H_2_O were 0.017, 1.67, 1.83, and 195, respectively. Approximately 1.0 g of KIT−6 was soaked with 1.25, 0.14, and 5.0 g of sucrose, H_2_SO_4_, and H_2_O, respectively. After a pre-carbonization in a tube furnace at 433 K for 6 h, the product was treated again by using a mixture of 0.8 g of sucrose, 0.09 g of H_2_SO_4_, and 5.0 g of H_2_O, and then subjected to the same pre-carbonization at 433 K for 6 h. Subsequently, the product was further carbonized in tubular furnace at 1173 K for 6 h under N_2_ protection. The silica was dissolved thrice in 3 mol/L of NaOH solution at 323 K. CMK−8 was obtained after filtration and then washed and dried at 373 K in an oven.

### 3.4. Preparation of the Modified GE

**Figure 7:**
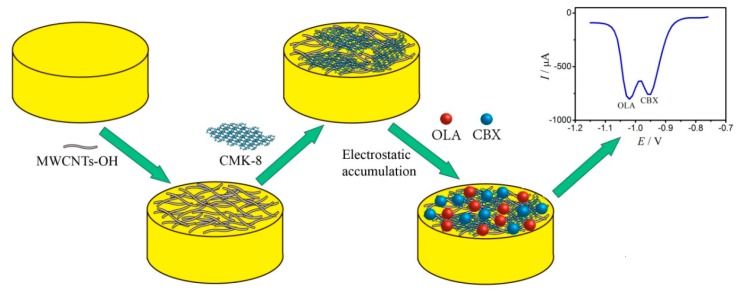
The schematic of the MWCNTs−OH/CMK−8/GE modified electrode’s fabrication and the detection of OLA and CBX.

The GE with a diameter of 3 mm was rinsed with double-distilled water, polished with 0.05 μm of γ-Al_2_O_3_, sonicated twice with double-distilled water, and air dried. Approximately 4 μL of the MWCNT−OH dispersion (4 mg/mL) was dropped onto the pretreated GE surface by using a microsyringe, dried under infrared lamp, and left at room temperature. Similarly, approximately 2 μL of the CMK−8 dispersion (4 mg/mL) was dropped onto the pretreated GE surface by using a microsyringe, dried under infrared lamp, and left at room temperature. Then, the modified MWCNTs−OH/CMK−8/GE was obtained. The schematics of the modified MWCNTs−OH/CMK−8/GE fabrication and the OLA and CBX detection are shown in Figure 7.

### 3.5. Electrochemical Test Method

An electrochemical test was performed by using the three-electrode system with 40-mL homemade electrolytic cell. Before the experiment, the freshly prepared modified electrode was scanned and stabilized by differential pulse voltammetry (DPV) in 0.6 mol/L of phosphate buffer saline (PBS) solution (pH = 7). The potential was set in the range of −0.4 V to −0.8 V, and the scan interval was set to 1 min. The determinant was added, and the time–current curve method was applied for enrichment. After the enrichment was completed, the three-electrode system was immediately transferred into a 0.6 mol/L solution of Na_3_PO_4_ and stirred for 1 s. DPV was conducted for the determination. 

### 3.6. Sample Treatment

A total of 8 g of pork samples (purchased from local market) were crushed by a cell grinder. Subsequently, appropriate OLA and CBX standard solutions and 10 mL acetonitrile solution were added. After 30 min of ultrasonic, the solution was centrifuged for 20 min at a centrifugal speed of 4000 RPM. The supernatant was dried with nitrogen and dissolved with 20 mL of a 0.6 mol/L PBS solution (pH = 7.0). The obtained solution was filtered by 0.22 μm filter membrane, and 1 mL sample solution was added to 19 mL PBS buffer for enrichment. After enrichment, the three-electrode system was immediately transferred to a 0.6 mol/L solution of Na_3_PO_4_ for detection by differential pulse voltammetry.

## 4. Conclusions

An electrochemical sensor modified by MWCNTs−OH/CMK−8/GE was fabricated in this study and successfully used in the simultaneous determination of OLA and CBX. The newly modified electrode demonstrated excellent electric conductivity and high catalytic activity. The results confirm that the modified electrode can perform individual and simultaneous determination of OLA and CBX with high sensitivity and selectivity. The fabricated sensor has a linear response at 0.2−500 nmol/L in optimum conditions, with LOD values of 104.1 and 62.9 pmol/L for OLA and CBX, respectively. As for individual determination, wide linear relationships were obtained for OLA at 0.05−500 nmol/L levels and CBX at 0.10−500 nmol/L levels, with LOD values of 20.7 and 50.2 pmol/L, respectively. Furthermore, OLA and CBX were successfully detected in the spiked pork samples by using the MWCNT−OH/CMK−8/GE electrode. Thus, the proposed modified electrode is a promising material for OLA and CBX determination in real samples.

## Figures and Tables

**Table 1:** Equivalent circuit parameters of OLA electro-reduction on differently modified electrodes obtained from Figure 3B.

Electrodes	*R*_s_ (Ω)	*R*_ct_ (Ω)	*Q*_dl_ (F)	*R*_ads_ (Ω)	*Q_asd_* (F)	*n*_1_	*n*_2_
GE	3.538	1.176 × 10^3^	6.209 × 10^−6^	1.456 × 10^5^	2.313 × 10^−5^	0.89	0.67
Error (%)	0.461	5.793	4.117	6.122	1.847	0.52	0.761
MWCNTs−OH/CMK−8/GE	3.132	5.884	1.175 × 10^−3^	—	1.816 × 10^−3^	0.81	0.95
Error (%)	0.740	8.89	10.31	—	9.73	2.178	2.223

**Table 2:** Real samples analysis (*n* = 5).

Title 1	Added (nM)	Recovery (%)	RSD (%)
OLA	1	96.10	8.18
	10	96.59	1.11
	100	107.78	6.71
CBX	1	96.10	8.18
	10	96.59	1.11
	100	107.78	6.71
